# (Fe-Co-Ni-Zn)-Based Metal–Organic Framework-Derived Electrocatalyst for Zinc–Air Batteries

**DOI:** 10.3390/nano13182612

**Published:** 2023-09-21

**Authors:** Anup Adhikari, Kisan Chhetri, Rajan Rai, Debendra Acharya, Jyotendra Kunwar, Roshan Mangal Bhattarai, Rupesh Kumar Jha, Dasharath Kandel, Hak Yong Kim, Mani Ram Kandel

**Affiliations:** 1 Central Department of Chemistry, Tribhuvan University, Kathmandu 44618, Nepal; adhika2@clemson.edu (A.A.); jyotendrakunwar@gmail.com (J.K.); 2 Department of Nano Convergence Engineering, Jeonbuk National University, Jeonju 561-756, Republic of Korea; debenaracharya88@jbnu.ac.kr (D.A.); khy@jbnu.ac.kr (H.Y.K.); 3 Department of Chemistry, Tri-Chandra Multiple Campus, Tribhuvan University, Kathmandu 44618, Nepal; rajanr@clemson.edu; 4 Department of Chemical Engineering, Jeju National University, Jeju 690-756, Republic of Korea; rshnmngl@gmail.com; 5 Kathmandu University, Dhulikhel 45210, Nepal; rupeshjha865@gmail.com; 6 Pulchowk Campus, Tribhuvan University, Kathmandu 44618, Nepal; kandeldasharath18@gmail.com; 7 Department of Chemistry, Amrit Campus, Tribhuvan University, Kathmandu 44613, Nepal

**Keywords:** zinc–air batteries, electrocatalysts, metal–organic frameworks (MOFs), energy density, energy storage and conversion

## Abstract

Zinc–air batteries (ZABs) have garnered significant interest as a viable substitute for lithium-ion batteries (LIBs), primarily due to their impressive energy density and low cost. However, the efficacy of zinc–air batteries is heavily dependent on electrocatalysts, which play a vital role in enhancing reaction efficiency and stability. This scholarly review article highlights the crucial significance of electrocatalysts in zinc–air batteries and explores the rationale behind employing Fe-Co-Ni-Zn-based metal–organic framework (MOF)-derived hybrid materials as potential electrocatalysts. These MOF-derived electrocatalysts offer advantages such as abundancy, high catalytic activity, tunability, and structural stability. Various synthesis methods and characterization techniques are employed to optimize the properties of MOF-derived electrocatalysts. Such electrocatalysts exhibit excellent catalytic activity, stability, and selectivity, making them suitable for applications in ZABs. Furthermore, they demonstrate notable capabilities in the realm of ZABs, encompassing elevated energy density, efficacy, and prolonged longevity. It is imperative to continue extensively researching and developing this area to propel the advancement of ZAB technology forward and pave the way for its practical implementation across diverse fields.

## 1. Introduction

### 1.1. Background on Zinc–Air Batteries 

The energy demand has increased as a result of rapid urbanization and technological innovation. In order to combat human-caused global warming and keep up with the need for energy, it is becoming increasingly important to design an eco-friendly energy environment [1]. Li-based batteries, Zn-based batteries, Na-based batteries, and supercapacitors make up the most well-known green energy storage systems (ESS) [2,3]. Notably, Zn-based batteries have drawn a significant amount of interest because of their efficient electrochemical behavior, their affordability, and the abundance of Zn metal relative to lithium. However, their potential for long-term use is restricted due to their inadequate energy density of approximately 250 Wh kg^−1^ [4,5]. Recently, researchers have been devoted to the synthesis of metal–air batteries due to their exceptional energy density, which produces electricity via metal–oxygen redox reactions in the atmosphere [6]. Zinc–air batteries have a history that goes back to the early 1800s, when experiments began exploring the use of zinc and oxygen for generating electricity [7,8]. In the 1960s, researchers dedicated their efforts to improving zinc–air batteries, which are known for their high energy density and their ability to be recharged by replacing the zinc anode. These batteries found practical applications in hearing aids, military devices, and more. Recent advancements in materials science have led to the improved performance and durability of these batteries [9]. Scientists are actively addressing challenges such as anode corrosion and limited recharge ability to make zinc–air batteries suitable for widespread use [10]. Zinc–air batteries have promising properties that make them appealing for use in electric cars, renewable energy storage, and a variety of other applications. These features include their high energy density, cost-effectiveness, and environmental friendliness [9]. Ongoing research aims to unlock their full potential and enhance their overall performance.

### 1.2. The Importance of Electrocatalysts in Zinc–Air Batteries

For zinc–air batteries to operate more effectively and efficiently, electrocatalysis is essential. There are numerous important reasons why electrocatalysts for zinc–air batteries are important. First, electrocatalysts speed up the conversion of oxygen molecules into hydroxide ions by facilitating the oxygen reduction process (ORR) at the cathode [9,11]. This enhancement in reaction efficiency leads to reduced energy losses during the ORR, thereby improving the overall battery performance. Bhardwaj et al. created a cubic CaCu_3_Ti_4_O_12_ perovskite electrocatalyst and used it as the air electrode in a Zn–air battery, as perovskite-based composites have been proven to improve the catalytic activity [12,13]. The material demonstrated a high specific capacitance of 801 mAh g^−1^ and remarkable cycling efficiency during charge–discharge cycles, with a power density of 127 mW cm^−2^ [12]. Additionally, electrocatalysts lower the over potential required for electrochemical reactions by reducing the activation energy barrier. This results in improved energy efficiency and increased voltage output of the battery [11,14]. Quian et al. prepared web-like interconnected porous carbon through the pyrolysis of NaCl/ZIF-8 composite and used it as the electrocatalyst in a ZAB. The composite exhibited a 6.6% higher output power (55.0 mW vs. 51.6 mW) in comparison to Pt/C. Further, the battery’s cycling life has been significantly enhanced and is now more than 140 h. This is 90 h more than Pt/C. The enhancement of performance can be attributed to excellent ORR activity and the stable discharge voltage of the composite [15]. To ensure the long-term functionality of zinc–air batteries that undergo repetitive charge and discharge cycles, electrocatalysts must exhibit excellent stability and durability [16]. A robust electrocatalyst can resist degradation and maintain its catalytic activity, thereby ensuring the prolonged performance and lifespan of the battery. Yang et al. created wrinkled MoS_2_/Fe-N-C nanospheres that were used as an ORR/OER electrocatalyst for a wearable Zn-air battery. The device had a specific capacitance of 442 mAh g^−1^ and an outstanding power density of 78 mW cm^−2^, with a cycle stability of 50 cycles at a current density of 5 mA cm^−2^. Here, Fe-N_4_ moieties combined with MoS_2_ particles contribute to lowering the energy barrier of ORR and OER. Furthermore, the Fe-N-C shell protects the MoS_2_ core from corrosion induced by alkaline electrolytes, which contributes to the device’s long life [17]. Moreover, electrocatalysts facilitate the oxygen evolution reaction (OER) at the cathode, allowing the efficient rechargeability of rechargeable zinc–air batteries [18,19,20]. By accelerating the OER, electrocatalysts minimize energy losses during the charging process, enabling efficient and effective recharging. Effective electrocatalysts significantly enhance the power density, energy density, and overall performance of zinc–air batteries. They promote faster reaction kinetics, improve cell efficiency, and increase the specific capacity of the battery, making them particularly advantageous for high-energy-demand applications such as electric vehicles and grid energy storage [21]. Li et al. employed a low-cost green approach to develop a CoN/CoFe/NC bifunctional electrocatalyst. The composite demonstrated outstanding electrocatalytic characteristics, with a low potential of 1.609 V for OER and a half-life potential of 0.89 V for ORR. The enormous number of reactive sites produced by the two-phase interface formed by coupling two structures can be attributed to the material’s exceptional OER and ORR performance. Furthermore, when employed as an air electrode in ZAB, the composite demonstrated an outstanding power density of 246 mW cm^−2^ and a constant voltage gap of 180 h [22]. Several studies have reported the capability of various MOF-based electrocatalysts to lower the overpotential and boost the stability, power density, and output voltage of the zinc–air battery [23,24,25,26]. In conclusion, electrocatalysts play a vital role in zinc–air batteries by enhancing reaction kinetics, reducing overpotential, ensuring stability, enabling rechargeability, and ultimately improving overall battery performance and efficiency. Continued research and development in electrocatalyst materials are crucial for further advancements in zinc–air battery technology.

### 1.3. Overview of Metal–Organic Frameworks (MOFs)

MOFs are crystalline materials made up of metal ions or clusters coordinated with organic ligands. They possess a porous structure with a large internal surface area, making them suitable for diverse applications. MOFs are synthesized through self-assembly, where metal ions or clusters bind with organic ligands to create extensive networks [27,28]. More than 20,000 MOF materials have reportedly been synthesized thus far, according to reports [29,30]. MOFs offer versatility at the molecular level, allowing researchers to design them with specific properties like pore size, surface chemistry, and thermal stability [27,31]. This adaptability enables customization for applications such as energy storage [32], separation [33], catalysis [34], and sensing [35]. The remarkable porosity of MOFs provides a substantial surface area for gas adsorption and storage, making them valuable for tasks like carbon capture and storage, as well as precise gas purification through selective separation [36]. In catalysis, MOFs act as platforms for organizing catalytically active sites, enhancing reaction efficiency by facilitating reactant accessibility. Their tunability also enables the incorporation of different metal species or clusters, leading to efficient and selective catalysts for specific chemical reactions. MOFs have further applications in drug delivery systems, utilizing their porous structure to encapsulate and deliver therapeutic agents with high loading capacities, controlled release kinetics, and targeted delivery [37]. Additionally, MOFs have been explored in sensing and detection, capitalizing on their selective adsorption capabilities to detect various analytes, including gases, volatile organic compounds, and heavy metal ions. By tailoring the MOF structure, their response to specific analytes can be customized, making them promising for sensor applications [38]. Overall, MOFs are a versatile and promising material class with wide-ranging applications in energy storage, gas storage, catalysis, drug delivery, and sensing. Continuous research and development in MOFs hold great potential for advancements and applications across industries [39,40].

### 1.4. Motivation for (Fe-Co-Ni-Zn)-Based MOFs-Derived Electrocatalysts

The motivation for employing electrocatalysts derived from Fe, Co, Ni, and Zn-based MOFs in zinc–air batteries can be attributed to several key factors. Firstly, Fe, Co, Ni, and Zn are abundant and widely available elements, making them cost-effective choices for electrocatalyst materials [41,42]. This utilization of abundant elements contributes to reducing the overall cost of the electrocatalyst, making it more economically feasible for large-scale production of zinc–air batteries. Moreover, materials derived from these MOFs exhibit high catalytic activity towards the ORR and OER occurring at the cathode and anode, respectively [43,44,45]. This enhanced catalytic activity facilitates the efficient and rapid conversion of oxygen molecules during discharge and recharge cycles, thereby improving the overall battery performance [46,47]. Tsai et al. developed a SAC (Fe, Ni, Zn)/NC bifunctional catalyst by anchoring a Fe-Ni-Zn triple single-atom catalyst (SAC) in an N-doped carbon structure. The material demonstrated excellent OER and ORR characteristics. Catalytic sites for OER and ORR reactions are provided by Fe-Nx, Zn-Nx, and Ni-Nx, and the synergetic combination of the three SAC in NC further enhances the catalytic activities. At 10 mA cm^−2^ current density, the composite displayed a half-wave potential of 0.88 V for ORR and a potential of 1.63 V for OER. A ZAB device with SAC (Fe-Ni-Zn)/NC as an air electrode demonstrated 809 mAh g^−1^ specific capacitance at 50 mA cm^−2^ and an outstanding power density of 300 mW cm^−2^. It additionally achieved cyclic stability of 2150 cycles over 358.3 h at 10 mA cm^−2^ current density [48]. MOFs derived from Fe, Co, Ni, and Zn offer tunable properties as they can be synthesized with different compositions and structures [49]. This tunability allows researchers to optimize the electrocatalyst’s performance by tailoring its structure, morphology, and composition to meet the specific requirements of zinc–air batteries, including activity, stability, and durability [50]. Additionally, MOFs derived from these metals often possess inherent structural stability, which is crucial for maintaining the electrocatalyst’s integrity and functionality during prolonged battery operation [51,52]. For instance, Gui et al. reported that a (Fe-Co)-based MOF-derived hybrid catalyst enhanced the performance of a zinc–air battery, catalyzing ORR and OER, resulting in high peak power density and a longer charge–discharge cycle [53]. Their robust structures enable them to withstand the demanding electrochemical conditions experienced during charge–discharge cycles, ensuring the long-term performance and lifespan of the energy storage system [54]. Furthermore, combinations of Fe, Co, Ni, and Zn-based MOFs as electrocatalysts can exhibit synergistic effects, where the presence of different metal species enhances catalytic activity and stability beyond what individual metals can achieve alone. This synergistic effect contributes to the improved overall performance and efficiency of zinc–air batteries. Jin et al. performed in situ carbonization of metal ion-absorbed PANI precursors, resulting in a composite with alloy nanoparticles encapsulated graphitic layer which is uniformly distributed in a N-doped carbon framework. The electrocatalyst demonstrated remarkable stability in ORR and OER processes. Furthermore, the electrocatalyst was used as an air electrode for flexible ZABs, and the assembled ZAB displayed a long cycling life of 22 h with an elevated power density of 125 mW cm^−2^ [55]. By incorporating Fe, Co, Ni, and Zn-based MOF-derived electrocatalysts into zinc–air batteries, researchers strive to develop cost-effective, high-performance, and durable energy storage systems. These electrocatalysts possess favorable properties such as abundance, catalytic activity, tunability, structural stability, and potential synergistic effects, making them highly promising candidates for advancing zinc–air battery performance and enabling practical applications in various fields, including portable electronics, electric vehicles, and renewable energy storage. Ren et al. synthesized a (Fe-Co-Ni)-based carbon nanorod hybrid and studied its catalytic application in a zinc–air battery. They reported that the synergetic effect of the combination of different metal components facilitated the accessibility of the reactants, resulting in an ORR half-wave potential of 0.84 V and an OER potential of 1.54 V at 10 mA cm^−2^. The zinc–air battery using the Fe-Co-Ni-based electrocatalyst exhibited a low voltage gap and longer durability [56]. Similarly, Li et al. reported a (Zn-Co-Fe)-tridoped-N-C nanocage as an efficient and stable electrocatalyst for the ORR in zinc–air batteries [57].

## 2. Synthesis Methods for (Fe-Co-Ni-Zn)-Based MOFs

### 2.1. Synthesis Techniques for (Fe-Co-Ni-Zn)-Based MOFs

**Figure 1:**
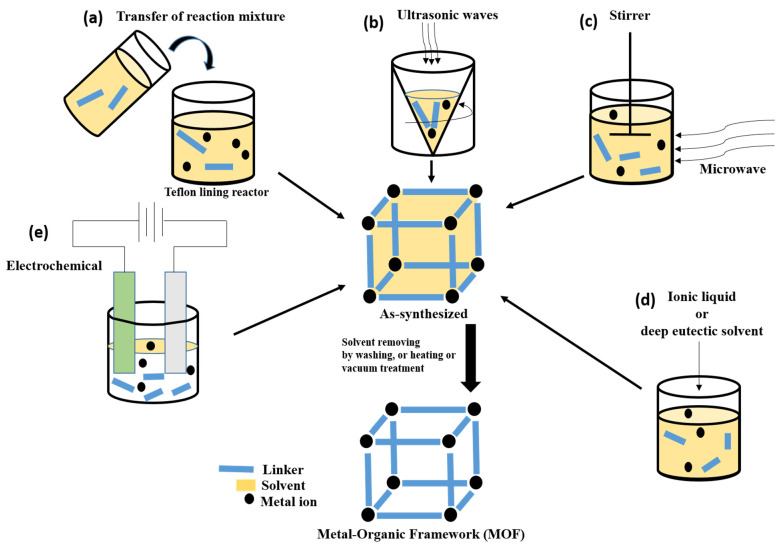
Some methods for MOF synthesis: (**a**) solvothermal or hydrothermal method; (**b**) ultra-sound assisted method; (**c**) microwave synthesis method; (**d**) ionothermal synthesis method; and (**e**) electrochemical synthesis method.

A variety of synthesis techniques are utilized to create Fe, Co, Ni, and Zn-based MOFs as electrocatalysts for energy storage and conversion applications. These techniques include solvothermal [58], hydrothermal [59], microwave-assisted [27], ultrasound-assisted [27], electrochemical [60], and ionothermal [61] methods, which are graphically represented in Figure 1. Solvothermal synthesis involves dissolving metal salts and organic ligands in a suitable solvent at high temperatures and pressures, followed by cooling to form MOF crystals. For instance, suitable solvents, such as N, N-dimethylformamide (DMF), or ethanol, are used to dissolve metal salts like nitrates, sulfates, chlorides, and organic linkers [58]. Lan et al. produced NiFe-MOF/NiFe_2_O_4_ spheres using a solvothermal technique and employed them as a bifunctional electrocatalyst for ORR/OER in ZABs. The synthesized composite outperformed pristine NiFe_2_O_4_ and NiFe-MOF in catalytic performance, indicating a synergetic combination of both. The resulting Zn-air battery has a power density of 158.4 mW cm^−2^ and a current density of 246.1 mA cm^−2^ [62]. Hydrothermal synthesis employs water as the solvent and promotes MOF crystal formation in a closed vessel under elevated temperatures and pressures [59]. In the experiment conducted by Lu et al., a bimetallic MOF framework (Co-Fe/Ni@HPA-MOF) was synthesized, and it exhibited excellent electrocatalytic behavior during OER [63]. Microwave-assisted synthesis utilizes microwave irradiation to rapidly heat the reaction mixture and accelerate MOF crystal formation. This approach necessitates considerably less reaction time compared to other established techniques. For example, the production of HKUST-1 utilizing microwave assistance in its synthesis, which has a chemical formula of [Cu_3_(BTC)_2_(H_2_O)_3_] (where BTC^3−^ = benzene-1,3,5-tricarboxylate), resulted in crystals that exhibited better yield and physical properties [59]. Ultrasound-assisted synthesis employs ultrasound waves to enhance the nucleation and growth of MOF crystals through acoustic cavitation. In an experiment conducted by Shahryari et al., a solution comprising Cu(NO_3_)_2_·5H_2_O and 2,6-pyridine dicarboxylic acid was prepared in double-distilled water. The mixture was subjected to ultrasound irradiation with a fixed frequency of 20 kHz for a duration of 20 min at a power output of 190 W and temperature maintained at 30 °C to yield Cu-MOF [59]. Electrochemical synthesis involves the use of electric current to drive the formation of MOF films or nanoparticles on conductive substrates. Mueller and colleagues conducted an academic inquiry into the characteristics of HKUST-1 that was produced via anodic dissolution. The method involved employing two copper plates submerged in a methanol solution of BTC, which were then subjected to a voltage range between 12 and 19 volts (resulting in a current flow of 1.3 amperes) for approximately 150 min. As revealed by their study, the resulting MOF exhibited significantly higher surface area compared to other synthesis approaches utilized for this purpose [60]. Ionothermal synthesis utilizes ionic liquids as reaction media, facilitating the synthesis of MOFs with enhanced stability and unique structures at high temperatures [61]. Azbell et al. have recently revealed that the employment of diverse linkers in conjunction with low-melting metal halide (hydrate) salts results in the production of exceptional-quality MOFs without requiring additional solvents. Frameworks synthesized using these ionothermal conditions exhibit porosities similar to those prepared through conventional solvothermal methods. Additionally, they reported the ionothermal synthesis of two frameworks that cannot be prepared directly under solvothermal conditions [60]. These synthesis techniques offer control over the composition, size, morphology, and crystallinity of Fe, Co, Ni, and Zn-based MOFs as electrocatalysts. The selection of a specific synthesis method depends on desired properties, scalability, and compatibility with energy storage and conversion applications. Ongoing research aims to develop innovative synthesis approaches to further enhance the performance and efficiency of MOF-based electrocatalysts.

### 2.2. Characterization Methods for MOFs-Derived Electrocatalysts

A variety of characterization techniques are used to analyze and comprehend the properties of electrocatalysts derived from MOFs. These methods provide valuable insights into these electrocatalysts’ structure, morphology, composition, surface chemistry, and electrochemical properties [64]. Commonly employed characterization methods for MOF-derived electrocatalysts include X-ray Diffraction (XRD), Scanning Electron Microscopy (SEM), Transmission Electron Microscopy (TEM), Energy-dispersive X-ray Spectroscopy (EDS), X-ray Photoelectron Spectroscopy (XPS), Fourier-transform infrared spectroscopy (FTIR), X-ray absorption spectroscopy (XAS), and various electrochemical techniques.

**Figure 2:**
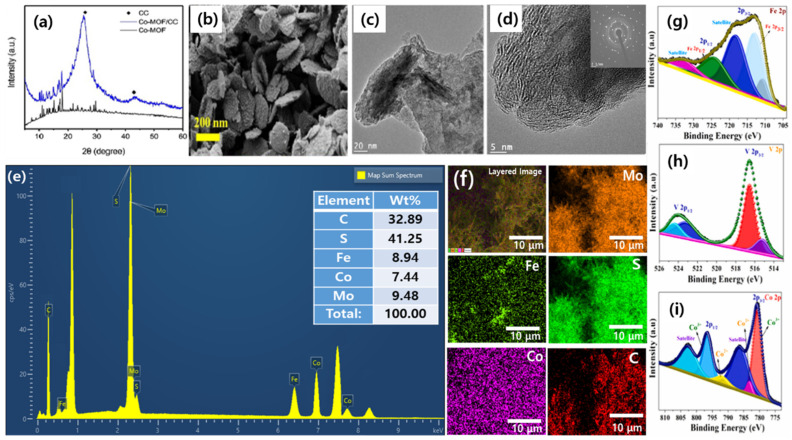
Characterization methods for MOF-derived electrocatalysts: (**a**) XRD patterns of the Co-MOF and Co-MOF/CC (reproduced with permission from [66]); (**b**) FESEM image of Fe-doped MOF CoV@CoO nanoflakes (reproduced with permission from [68]); (**c**–**f**) TEM images with SAED pattern (inset; scale is 2 1/nm), EDX spectrum, and elemental mapping of FeS_2_-MoS_2_@CoS_2_-MOF, respectively (reproduced with permission from [69]); and (**g**–**i**) XPS spectra of Fe 2p, V 2p and Co 2p, respectively, in Fe-doped MOF CoV@CoO nanoflakes (reproduced with permission from [68]).

XRD is utilized to determine the crystal structure and phase purity of MOF-derived electrocatalysts. It provides information about the arrangement of atoms in the material and enables the identification of different crystalline phases present [65]. For instance, Ao and colleagues created a MOF-derived sulfide-based electrocatalyst that displayed a distinct phase of pure crystallinity in its XRD pattern (Figure 2a) [66].

SEM allows for the visualization of the surface morphology and particle size of the electrocatalyst. It offers high-resolution images and can uncover the presence of agglomerates or surface defects [67]. As an illustration, the SEM images of iron-doped cobalt- vanadate- cobalt oxide with metal–organic framework-oriented nanoflakes synthesized by Muthurasu and researchers demonstrated surface morphology, its thickness, and its layered structure of Fe-doped MOF CoV@CoO nanoflakes (Figure 2b) [68].

TEM provides detailed information about the internal structure and morphology of MOF-derived electrocatalysts at the nanoscale. It enables the determination of particle size, shape, distribution, and the presence of defects or dislocations [64]. To give an idea, the TEM image of integrated bimetallic sulfide-coupled 2D MOF-derived mesoporous CoS_2_ nanoarray hybrids, developed by Chhetri and researchers showed heterointerfaces of FeS_2_, MoS_2_, and CoS_2_ with their respective interplanar spaces (Figure 2c,d) [69]. EDS is employed to analyze the elemental composition of the electrocatalyst. By detecting the characteristic X-rays emitted by the elements in the material, it provides quantitative information about their relative concentrations [70]. The presence of Co, Mo, Fe, C, N, and S in the nanoflakes was confirmed by EDS, in the work done by Chhetri and researchers (Figure 2e,f) [69].

XPS is employed to determine the elemental composition and chemical state of the electrocatalyst’s surface. It provides information about the oxidation states of the elements and can detect surface contaminants or adsorbates [71]. The XPS study of iron-doped cobalt- vanadate- cobalt oxide with metal–organic framework-oriented nanoflakes synthesized by Muthurasu and researchers provided the idea of the oxidation state of the resulting materials. XPS clearly demonstrated the variable oxidation state of Co, Fe, O, N, and V (Figure 2g–i) [68].

FTIR is a common characterization method used to get an infrared spectrum of absorption or emission of a solid, liquid, or gas. FTIR helps to identify organic, polymeric, and, in some cases, inorganic materials. The FTIR analysis method uses infrared light to scan test samples and observe chemical properties [69,72]. Additionally, XAS is a commonly used technique to examine atomic local structure as well as electronic states. Usually, an X-ray strikes an atom and excites a core electron that can either be promoted to an unoccupied level or ejected from the atom [73]. It can interpret the properties of catalytic materials in situ and operand. XAS can be divided into X-ray absorption near edge structure (XANES) and the extended X-ray absorption fine structure (EXAFS). XANES delivers evidence on the electronic structure of the catalyst, while the EXAFS evaluates the interatomic distances and coordination numbers of the atoms in the electrocatalyst [74].

Various electrochemical techniques, such as cyclic voltammetry, linear sweep voltammetry, chronoamperometry, chronopotentiometry, electrochemical surface area (ECSA), and electrochemical impedance spectroscopy, are utilized to assess the electrochemical activity, stability, and performance of MOF-derived electrocatalysts. These techniques offer information about catalytic activity [75], reaction kinetics [76], and charge transfer processes occurring at the electrode–electrolyte interface [76]. By employing a combination of these characterization methods, researchers can gain a comprehensive understanding of the morphological, compositional, and electrochemical properties of MOF-derived electrocatalysts [51]. This knowledge aids in the optimization and design of efficient and stable electrocatalytic materials.

## 3. Electrocatalytic Properties of (Fe-Co-Ni-Zn)-Based MOFs-Derived Electrocatalysts

The electrocatalysts derived from (Fe-Co-Ni-Zn)-based MOFs exhibit a range of electrocatalytic properties that hold promise for diverse applications. These properties encompass their performance in key reactions such as the ORR and OER, which are critical for efficient energy conversion in fuel cells and metal–air batteries [77,78]. The electrocatalysts demonstrate notable ORR activity by facilitating the conversion of oxygen molecules into hydroxide ions at the cathode [79]. Similarly, they exhibit excellent OER activity by promoting the oxidation of water molecules, generating oxygen gas during the charging process in energy storage devices [80]. The catalytic efficiency of these MOFs-derived electrocatalysts is high due to their distinctive composition and structure, leading to reduced overpotential requirements for electrochemical reactions. Consequently, this enhances energy efficiency and overall performance in electrochemical devices. Notably, the electrocatalysts based on Fe, Co, Ni, and Zn elements offer exceptional stability and durability, enabling them to maintain catalytic activity even under demanding operating conditions, ensuring the long-term performance and lifespan of electrochemical devices [81,82,83]. The composition and structure of (Fe-Co-Ni-Zn)-based MOFs-derived electrocatalysts can be precisely tuned to achieve desired properties. Through the combination of different metal ions or clusters, synergistic effects can be harnessed, resulting in enhanced catalytic performance surpassing that of individual metals alone [84]. These electrocatalysts also exhibit selectivity in catalytic reactions, enabling precise control over the desired products. They can promote specific chemical transformations while minimizing undesired side reactions, rendering them suitable for applications requiring high selectivity, such as in the pharmaceutical and fine chemical industries [85]. Moreover, MOF-derived electrocatalysts possess a highly porous structure, providing a large surface area for electrochemical reactions [86,87]. This increased surface area enhances reactant accessibility to catalytic sites, thereby improving reaction kinetics and overall efficiency. The exceptional electrocatalytic properties of (Fe-Co-Ni-Zn)-based MOFs-derived electrocatalysts, including high ORR and OER activity, catalytic efficiency, stability, tunability, selectivity, and large surface area, position them as attractive candidates for various energy storage and conversion applications such as fuel cells, metal–air batteries, and electrolyzers [88]. Further research and development efforts in this domain are crucial for optimizing their electrocatalytic performance and expanding their practical applications.

## 4. Performance in Zinc–Air Batteries

Zinc–air batteries are appealing for a variety of applications due to their favorable characteristics such as high energy density, affordability, and eco-friendliness [89]. The performance of zinc–air batteries is evaluated based on several key factors. Energy density is a crucial measure, representing the amount of energy stored per unit weight or volume of the battery [90]. Zinc–air batteries possess comparable or even superior energy density to lithium-ion batteries, making them suitable for power-hungry devices like electric vehicles and portable electronics [91]. Specific energy and specific power are important considerations, indicating the amount of energy stored per unit weight and the rate at which the energy can be delivered, respectively [92]. Zinc–air batteries typically demonstrate high specific energy, enabling substantial energy storage [93]. However, their specific power is generally lower compared to other battery technologies, which can restrict their performance in applications requiring high power output [94]. Zinc–air batteries offer a relatively high cell voltage, typically ranging from 1.2 to 1.4 volts, which is advantageous for many applications [95]. Traditionally, zinc–air batteries have been non-rechargeable, meaning they cannot be recharged once depleted. However, ongoing research is focused on developing rechargeable zinc–air batteries by addressing challenges such as dendrite formation, electrolyte stability, and electrode degradation [10]. The lifespan of a zinc–air battery depends on various factors, including electrode materials, electrolyte composition, and operating conditions. Under optimal circumstances, zinc–air batteries can exhibit a long lifespan, making them suitable for applications requiring prolonged use [10,94]. Additionally, zinc–air batteries with suitable electrolytes have an extended shelf life, allowing them to be stored for extended periods without significant self-discharge [96]. Zinc–air batteries are environmentally friendly as they utilize abundant and non-toxic zinc as the anode material [97]. Zinc is readily available, making it a cost-effective and sustainable choice. Moreover, zinc–air batteries produce no greenhouse gas emissions during operation [98]. Despite their advantages, zinc–air batteries face challenges such as limited rechargeability, relatively lower specific power, and the need for improved electrolyte stability [99]. Ongoing research and development endeavors aim to overcome these limitations and enhance the performance of zinc–air batteries, making them more competitive in applications that require high energy density and cost-effectiveness. Table 1 summaries electrocatalytic and ZAB performances shown by oxygen electrocatalysts of (Fe-Co-Ni-Zn)-based MOF materials in recent years.

### 4.1. Integration of (Fe-Co-Ni-Zn)-Based MOFs-Derived Electrocatalysts in Zinc–Air Batteries

The catalytic properties of MOFs have been extensively studied with various transition metals, such as iron, cobalt, nickel, and zinc [116]. These metals are capable of producing stable MOFs using organic ligands that accompany excellent redox activity and enhance ORR performance [117]. Hence, they are suitable for electrochemical reactions in the formation process of zinc–air batteries. The synthesis route for creating metal-based catalysts using Fe/Co/Ni/Zn involves mixing the two components, metal ions, and linker, under appropriate conditions so that porous crystalline structures are obtained [118,119].

**Figure 3:**
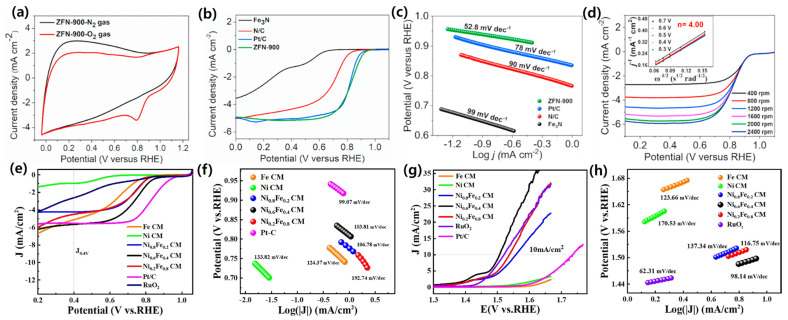
(**a**) CV curves of ZFN-900 in N_2_- and O_2_-saturated 0.1 M KOH solution with a scan rate of 100 mV s^−1^; (**b**) ORR polarization curves of different catalysts at 1600 rpm; (**c**) Tafel slopes of different catalysts derived from ORR LSV curves; (**d**) LSVs for ZFN-900 at various rotating speeds, inset is the corresponding K−L plot (reproduced with permission from [26]); (**e**) LSV; (**f**) Tafel slopes of samples for ORR; (**g**) LSV; and (**h**) Tafel slopes of samples for OER (reproduced with permission from [100]).

Radwan et al. synthesized a new Fe_3_N embedded in 3D high surface area ZIFs as a precursor to producing nitrogen-doped carbon network encapsulated Fe_3_N (ZFN-900), and then employed it as a low-cost and efficient electrocatalyst for ORR and ZABs. Such ZFN-900 has noticeable activity in an O_2_-saturated electrolyte with the cathodic current peak at ≈0.80 V, compared to that within a N_2_-saturated solution, signifying a promising ORR electrocatalytic performance (Figure 3a). The remarkable ORR performance for ZFN-900 was examined, showing a significant half-wave potential (E_1/2_) of 0.85 V than that of Pt/C (0.82 V) and N-doped carbon (0.71 V) (Figure 3b). As presented in Figure 3c, the Tafel slope for ZFN-900 is 53 mV dec^−1^, lesser than that for Pt/C (78 mV dec^−1^), N-doped carbon (90 mV dec^−1^), and Fe_3_N (99 mV dec^−1^), signifying quicker reaction kinetics in ZFN-900. Figure 3d inset shows the n value to be ≈4.0 in a potential window of 0.3–0.7 V, signifying first-order reaction kinetics, four-electron pathway of ORR for ZFN-900 [26]. Similarly, Li et al. developed MOF-derived Ni-Fe alloys embedded graphitic carbon electrocatalyst with a smaller voltage gap (0.76 V) than that for commercial Pt/C and RuO_2_ and a power density of 59.83 mW cm^−2^ for ZAB performance. Likewise, Li and a team of researchers developed a bifunctional electrocatalyst via Fe/Ni-based MOF following carbonization. As shown in Figure 3e,f, LSV of Ni_x_Fe_1−x_CMs (0 < x < 1) showed significant ORR performance compared to single metal element-based carbon materials (SMCMs), and lower Tafel slopes also verifies the synergetic effect of two metals for electrocatalytic properties of double metal elements-based carbon materials (DMCMs). The OER LSV of Ni_x_Fe_1−x_ CMs (0 < x < 1) showed remarkable OER performance as compared to RuO_2_, and DMCMs displayed lower Tafel slope than SMCMs (Figure 3g,h) [100].

**Figure 4:**
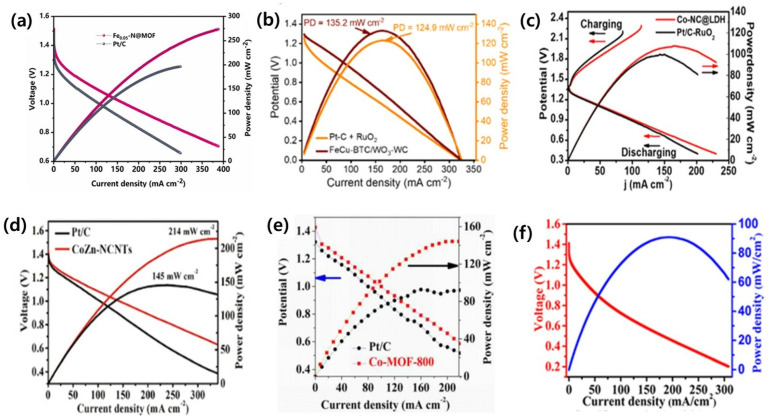
(**a**) Polarization curve and power density plot of Zn-air fuel cell with the Fe0.05-N@MOF and Pt/C as cathode (reproduced with permission from [120]); (**b**) FeCu-BTC/WO_3_-WC and Pt-C+ RuO_2_ (reproduced with permission from [20]); (**c**) Co-NC@LDH and Pt/C-RuO (reproduced with permission from [25]); (**d**) CoZn-NCNTs and Pt/C + RuO (reproduced with permission from [105]); (**e**) Co-MOF-800 and Pt/C (reproduced with permission from [107]); and (**f**) FeS/Fe_3_C@NS-C-900 (reproduced with permission from [108]).

Zhang et al. developed porous Fex-N@MOF as a highly efficient catalyst for oxygen reduction over a wide pH range with a maximum power density of ca. 274 mW cm^−2^, which is approximately 28% greater than that of Pt/C (196 mW cm^−2^) in (Figure 4a) [120]. Paudel et al. functionalized metal nodes–organic bridge ligands that were integrated on polyvalent tungsten oxide–carbide interfaces with highly porous nano-dendritic architecture. Thus, the formed FeCu-BTC/WO-WC showed a power density of 135.2 mW cm^−2^ (Figure 4b) for ZAB [20]. Similarly, Chen et al. synthesized a composite (Co-NC@LDH) by anchoring NiFe-layered double hydroxide (NiFe-LDH) nanosheets on the surface of a ZIF-derived carbon-based framework. It showed a high peak power density (107.8 mW cm^−2^) (Figure 4c) and excellent durability (over 300 h) when used in ZAB [25]. Liu and a team of researchers constructed (CoZn-NCNTs) composite by Co nanoparticles embedded in like N-doped carbon nanotubes, which showed a high-power density of 214 mW cm^−2^ (Figure 4d) with a voltage retention of 99.4%, in ZAB [105]. Likewise, Duan et al. constructed novel Co-MOF, O-doped carbon (Co-MOF-T) based on Zn, Co-doped glucosamine, and ZIF-8. The primary Zn–air batteries using a Co-MOF-800 air electrode achieved a high open-circuit voltage of 1.38 V, a specific capacity of 671.6 mAh g^−1^, and a prominent peak power density of 144 mW cm^−2^ (Figure 4e) [107]. Furthermore, Li and co-authors synthesized FeS/Fe_3_C nanoparticles embedded in a porous N, S-dual doped carbon honeycomb-like composite (FeS/Fe_3_C@NS-C-900). It demonstrated a power density of 90.9 mW cm^−2^ (Figure 4f), a specific capacity of 750 mAh g^−1^, and cycling stabilities over 865 h (1730 cycles) at 2 mA cm^−2^ for rechargeable ZAB [108].

**Figure 5:**
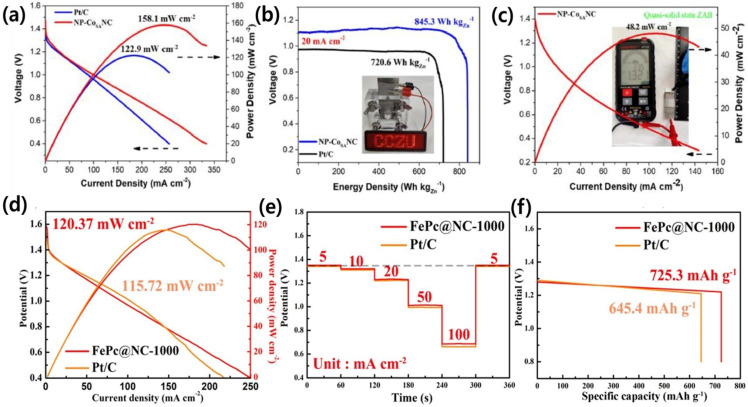
(**a**) Corresponding power density curves of aqueous primary ZABs for NP-CoSANC and Pt/C; (**b**) discharge curves of aqueous primary ZABs for NP-CoSANC and Pt/C (inset: photograph of LED screen powered by an aqueous primary ZAB); (**c**) long time discharge/charge cycle testing of two aqueous primary ZABs with NP-CoSANC catalyst and Pt/C + RuO_2_ catalyst [121]; and (**d**–**f**) polarisation and power density profiles, rate capacity, and mass-specific capacity of FePc@NC-1000 and Pt/C, respectively [122].

Rong et al. prepared a two-dimensional nanoporous Co single-atom decorated nitrogen-doped carbon catalyst (NP-Co_SA_NC) by the pyrolysis of urea-adsorbed Co-TPyP MOF precursor, which displays the peak power density of 158.1 mW cm^−2^ at the current density of 249.0 mA cm^−2^, exceeding that of Pt/C + RuO_2_ (Figure 5a). Additionally, at the current density of 20 mA cm^−2^, the aqueous primary ZAB with NP-Co_SA_NC air cathode shows a higher voltage platform than that of Pt/C + RuO_2_ air cathode (Figure 5b). Interestingly, an aqueous primary ZAB with NP-Co_SA_NC air cathode can steadily light up a LED panel, suggesting its real-time application and favorable prospects in power devices (inset in (Figure 5b)). The quasi-solid-state ZAB shows a high open circuit voltage of 1.321 V under a flat state in ambient air (inset in (Figure 5c)) [121]. Moreover, Dong et al. reported that FePc@NC-1000 delivered a higher power density of 120.37 mW cm^−2^ compared to Pt/C, indicating good mass transfer kinetics (Figure 5d). As portrayed in Figure 5e, the ZABs upheld a relatively steady output voltage from 5 to 100 mA cm^−2^. Furthermore, upon returning the current density to 5 mA cm^−2^, the discharge voltage of the ZABs resumed to its initial value, indicating high rate capability and durability. The specific capacity of the ZAB based on FePc@NC-1000 (725.3 mAh g^−1^) is significantly higher than that of Pt/C (645.4 mAh g^−1^) discharged at 10 mA cm^−2^ (Figure 5f). Overall, the FePc@NC-1000 proves to be an appropriate Pt-free cathode catalyst with significant applications [122].

As scientific exploration progresses toward the enhancement and refinement of electrocatalyst composition and fabrication, encouraging progress is foreseen in creating sustainable energy storage solutions that are enduring, proficient, and environmentally benign. The incorporation of electrocatalysts derived from MOF material into ZABs holds optimistic potential for establishing dependable high-capacity energy-storing mechanisms.

### 4.2. Comparison with Other Electrocatalysts

For ZABs to become a viable and sustainable source of energy storage, various criteria must be taken into account in the selection of electrocatalysts. These factors include catalytic efficiency, cost-effectiveness, scalability, stability, and availability [123]. Current studies are focused on advancing research efforts toward designing efficient electrocatalysts aimed at promoting extensive usage of these batteries as an eco-friendly solution for long-term energy storage needs. Numerous categories of electrocatalysts have been investigated for their viability in ZAB usage, with the primary function being to promote ORR and OER. The role played by these electrocatalysts is pivotal.

In comparison to commercial non-porous noble metal catalysts, MOF-based catalysts hold several distinct advantages [124]. Firstly, they enable the simple incorporation of highly dispersed hetero atoms such as metal atoms and N, O, S, and P, which can customize the local electronic configuration of the catalyst; this reduces the adsorption energy of intermediate species, leading to enhanced catalytic performance. Secondly, controlling size, morphology, and pristine structure help improve that performance. Thirdly, conductive ligands impart good electrical conductivity within MOFs, ensuring prompt electron transfer. Fourthly, their supramolecular attributes provide superior electrochemical stability and durability in electrocatalysis applications [125], hence, crucially making it essential to develop MOFs with proper organic ligand architecture characteristics containing appropriate accessible voids along with suitable metallic ions for productive MOF-based catalysis reactions.

## 5. Strategies for Enhancing Performance

To enhance the performance of catalysts in zinc–air batteries, various strategies can be employed. These strategies involve designing catalysts with precision, modifying their surface properties [126], utilizing nanostructures [127], incorporating alloying [128] and hybrid materials [129], employing porous catalyst supports [86], ensuring catalyst stability [81], optimizing the interface between the catalyst and electrolyte [130], utilizing advanced characterization techniques, and optimizing the integration of the catalyst into the battery system. By implementing these strategies, the goal is to improve catalytic activity, enhance charge transfer kinetics, increase durability, and facilitate efficient mass transport [88]. Ultimately, these enhancements result in improved energy efficiency, higher power output, and a longer lifespan for zinc–air batteries.

### 5.1. Doping and Alloying Approaches

**Figure 6:**
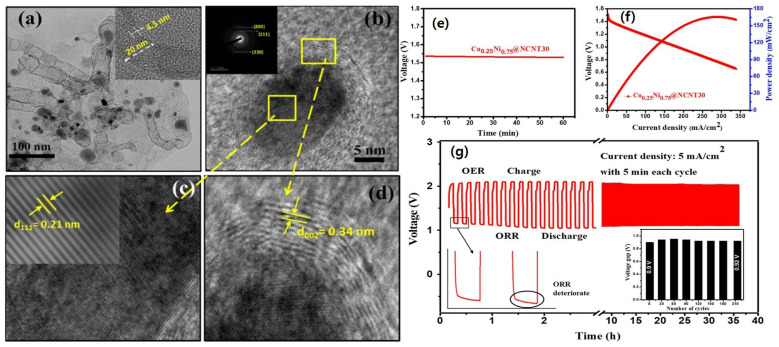
(**a**) TEM and (**b**–**d**) HRTEM images of Co_0.25_Ni_0.75_@NCNT30. Inset (**a**) highlights the HRTEM image of a chosen NCNT, while insets (**b**) and (**c**) exhibit SAED and inverse Fast Fourier-transform (FFT) patterns, respectively (Scale of inset of (**b**) is 10 1/nm); (**e**) Co_0.25_Ni_0.75_@NCNT30-based ZAB is characterized by open-circuit voltage; (**f**) discharge polarization curve and power density plot; and (**g**) galvanostatic charge–discharge cycling performance. A magnified section of the discharge profile (initial two cycles) is portrayed in the left inset of (**g**); and the right inset features the plot of charge–discharge voltage gap versus cycle number (reproduced with permission from [109]).

Doping heteroatoms into the catalyst materials is a technique used to boost the performance of zinc–air batteries. By incorporating heteroatoms like nitrogen, carbon, or sulfur into the catalyst’s structure, various enhancements can be achieved [131,132]. The presence of heteroatoms alters the catalyst’s electronic structure, surface chemistry, and catalytic activity, resulting in improved ORR and OER capabilities [132]. Heteroatom doping enhances the electrocatalytic activity, increases the number of active sites, and accelerates the overall reaction kinetics [133]. For example, nitrogen doping in carbon nanotubes has been shown to significantly enhance their electrocatalytic activity for ORR in fuel cells, due to the introduction of nitrogen heteroatoms modifying the electronic structure of carbon nanotubes, creating additional active sites for oxygen adsorption and facilitating the transfer of electrons during the ORR [134]. This approach holds great promise for optimizing zinc–air batteries, leading to enhanced energy efficiency and prolonged battery life [12]. Huang et al. synthesized porous carbon doped with Co, Ni, N, and Zn using a one-pot technique. With a half-wave potential of 0.864 V, a low current density of 6.40 mA cm^−2^, and an excellent cycle life, the porous multi-doped carbon demonstrated remarkable ORR performances. The porous structure, Co-N reactive sites, and synergetic combination of different dopant atoms can be attributed to the excellent ORR performance of Ni-Co-Zn-N-PC. Similarly, ZABs with Ni-Co-Zn-N-PC cathodes demonstrated exceptional capacity, power density, and endurance [135]. Kundu et al. synthesized hierarchical hollow Co_0.25_Ni_0.75_@NCNT for OER/ORR/ZAB (Figure 6) cycles [109]. The analysis using TEM, HRTEM, and SAED revealed the presence of a hierarchical hollow structure with lattice fringes spacing of 0.21 and 0.34 nm for CoNi alloy and graphitic carbon, respectively (Figure 6a–d). Figure 6e shows that our ZAB (zinc–air battery) has an open-circuit voltage of approximately 1.53 V. Furthermore, the ZAB based on Co_0.25_Ni_0.75_@NCNT30 demonstrated a peak power density of around 167 mW cm^−2^ (Figure 6f). To assess the cycling stability, galvanostatic charge–discharge measurements were conducted at a current density of 5 mA cm^−2^ (Figure 6g). The results confirmed that the device remains rechargeable for 36 h with an initial voltage gap of about 0.9 V. Throughout 215 cycles, there was only a slight increase of approximately 20 mV in the voltage gap (Figure 6c, inset). The initial voltaic efficiency was measured at 56.3%, and after 36 h of long charge–discharge cycles, the voltaic efficiency experienced only a marginal loss of around 1.6%, demonstrating excellent stability and rechargeability of our ZAB. Upon meticulous analysis of the charge–discharge profile, as depicted in Figure 6c, it becomes readily apparent that the initial voltage manifests at approximately 1.5 V, in consonance with the discharge polarization curve delineated in Figure 6f. A more granular examination of the segment of the discharge profile, as illustrated in Figure 6g (inset), elucidates a discernible attenuation in oxygen reduction reaction (ORR) activity. This diminution is likely attributed to the incipient deterioration of the catalyst and the concurrent corrosion of the carbon support, phenomena frequently observed in the context of alternating charge–discharge cycles, consistent with analogous observations made with alternative catalyst formulations. Noteworthy, however, is the observation that the ORR activity of the catalyst in question does not succumb to substantial degradation, as is evidenced by the sustained charge–discharge profile over protracted cycling intervals. Manifesting a mere marginal alteration of approximately 20 mV in the voltage gap after an exhaustive 36 h cycling period, the catalyst’s performance remains commendably stable. Furthermore, it merits consideration that the discharge voltage exhibited by our catalyst modestly trails that of the reference zinc–air battery (ZAB). This phenomenon may be ascribed, at least in part, to the engagement of a two-electron pathway for the oxygen reduction process, introducing an intricacy into the electrochemical landscape.

Alloying metal is a method employed to enhance the electrocatalyst performance in zinc–air batteries. By blending different metals within the catalyst, a range of improvements can be achieved. Alloying modifies the catalyst’s electronic structure and surface characteristics, resulting in enhanced oxygen reduction and evolution reactions. It also reinforces the catalyst’s structure, increasing its durability and preventing degradation [136]. Furthermore, alloying enhances the electrochemical stability of the catalyst, enabling reliable performance even in demanding conditions. Overall, incorporating alloyed metals in the electrocatalyst significantly boosts the efficiency, energy conversion, and overall effectiveness of zinc–air batteries.

### 5.2. Structural Modifications of MOFs-Derived Electrocatalysts

**Figure 7:**
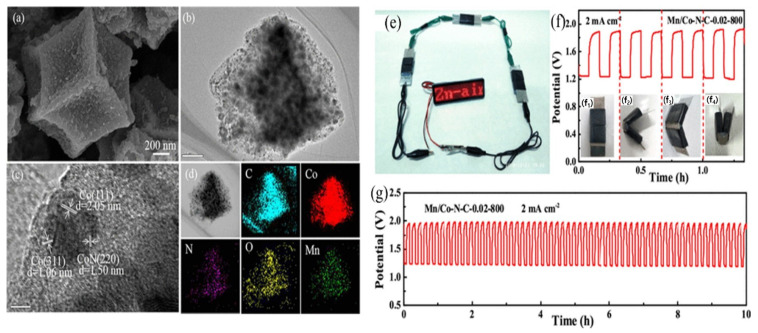
(**a**) FESEM image; (**b**,**c**) HRTEM image (scale bar of (**b**) is 200 nm and (**c**) is 5 nm); (**d**) elemental mapping analysis of Mn/Co-N-C-0.02-800; (**e**) visual manifestation of practical implementation is provided in through a photographic representation depicting a LED aglow, orchestrated by an interconnected sequence of three all-solid-state ZABs; (**f**) cyclic stability of all-solid-state Mn/Co-N-C-0.02-800 ZAB tests under different bending states (subfigures (**f_1_**–**f_4_**)) as presented at 2 mA cm^−2^ with per cycle period of 10 min; (**g**) galvanostatic discharge-charge cycling behavior of the Mn/Co-N-C-0.02-800 all-solid-state zinc–air battery (reproduced with permission from [111]).

Structural modifications are utilized to enhance the performance of electrocatalysts derived from MOFs based on Fe, Co, Ni, and Zn [137]. These modifications involve altering the composition, morphology, and architecture of the electrocatalysts. One common approach is the inclusion of dopants, such as heteroatoms or different elements, to improve electrocatalytic activity and stability [126]. This adjustment of the electronic properties enhances active sites and facilitates efficient charge transfer. Surface functionalization is another strategy where the MOFs-derived electrocatalysts are modified with functional groups or active sites to enhance their interaction with reactant molecules and catalytic performance [138]. This can be achieved through the addition of organic ligands, metal complexes, or nanoparticles on the surface [139]. For instance, Poudel et al. functionalized metal nodes–organic bridge ligands integrated on polyvalent tungsten oxide–carbide interfaces with highly-porous nano-dendritic architecture. Thus, formed FeCu–BTC/WO-WC demonstrated a half-wave potential of 0.81 V (very similar to benchmark Pt-C) along with an elevated power density of 135.2 mW cm^−2^ for ZAB [20]. Nanostructuring techniques involve controlling the size, shape, and distribution of nanoparticles within the MOF structure. This nanostructuring enhances the surface area, facilitates mass transport, and improves reactant accessibility to catalytic sites [140]. Liu and a group of scientists fabricated a composite material by embedding Co nanoparticles in N-doped carbon nanotubes. This resulted in an exceptional power density value of 214 mW cm^−2^ for ZAB, with superior stability (only 11 mV negative shift for 5000 cycles). It is much higher than the benchmark catalysts, respectively, derived from zinc- and cobalt-based MOF precursors [105]. Hierarchical structures can also be created by combining MOFs with other materials or incorporating secondary templates, improving mass transport, active site utilization, and catalytic performance by offering multiple porosity length scales [86]. Xu et al. engineered hierarchical MOF, Fe_3_O_4_/Fe-N-C-CNT (FeNC) using Fe^3+^ adsorbed ZIF-8 as a chemical precursor. FeNC displayed remarkable ORR capabilities, endurance, and specificity compared to the conventional Pt/C electrocatalyst under alkaline conditions. When employed as an air electrode in a primary zinc–air battery, it exhibits a superior peak power density of 118.05 mW cm^−2^ than that observed with a Pt/C cathode while also exhibiting faster reaction kinetics [141]. Metal cluster engineering allows for the precise control of catalytically active sites by designing MOFs with specific metal clusters or nanoparticles, resulting in enhanced catalytic activity, selectivity, and stability [142]. In 2020, Li and co-authors synthesized FeS/Fe_3_C nanoparticles embedded in a porous N, S-dual doped carbon honeycomb-like composite (FeS/Fe_3_C@NS-C-900). This metal cluster demonstrated a power density of 90.9 mW cm^−2^, and cycling stabilities over 865 h at 2 mA cm^−2^ for rechargeable ZAB [108]. Defect engineering involves the intentional introduction of controlled defects or vacancies in the MOF structure to create additional active sites and modify electronic properties, leading to improved catalytic activity, charge transfer, and facilitating specific reactions [143]. Zhao et al. manufactured carbon fibers with natural carbon imperfections (D-CFs) by introducing heteroatoms through seaweed polysaccharide precursor fabrication, followed by de-doping. The ORR electrocatalyst capability of D-CFs resulted in an initial potential of 0.92 V while the peak power density observed from zinc–air batteries was notably higher at 238 mW cm^−2^ than those made from commercially available Pt/C materials which had only managed a reading of about 154 mW cm^−2^ [144]. Similarly, Wei et al. synthesized Mn/Co-N-C as a highly effective dodecahedral nanocage electrocatalyst for ORR/zinc–air batteries (Figure 7a–d) [111]. This investigation entailed an exploration into the efficacy and versatility of the Mn/Co-N-C-0.02-800 electrocatalyst through the construction of all-solid-state zinc–air batteries employing an alkaline poly(vinyl alcohol) electrolyte. The assembled battery exhibited an impressive open circuit potential of 1.39 V and successfully powered an LED viewing screen using three batteries in series (Figure 7e). Moreover, the battery’s flexibility was tested by bending it into different shapes, yet it maintained a stable charge (1.92 V) and discharge (1.21 V) at 2 mA cm^–2^ potentials even at various angles (Figure 7f). The Mn/Co-N-C-0.02-800 cathode displayed excellent first charge and discharge potentials of 1.95 V and 1.23 V, respectively, and showcased superb cycling stability after 60 charging and discharging cycles (Figure 7g). These results demonstrate the potential of the catalyst for use in high-performance, flexible, and stable all-solid-state zinc–air batteries due to the synergism of manganese dopant and Co-N-C in the Mn/Co-N-C.

Thus, structural modifications enable the customization and optimization of Fe, Co, Ni, and Zn-based MOFs-derived electrocatalysts for various electrochemical applications. By tailoring the molecular and nanoscale structure, researchers can achieve improved catalytic performance, stability, selectivity, and efficiency, making these electrocatalysts highly suitable for energy storage, conversion, and other electrochemical processes.

### 5.3. Surface Engineering and Catalyst Support Strategies

To enhance the performance of zinc–air battery electrocatalysts, surface engineering techniques, and catalyst support strategies are employed [145]. These methods involve modifying the surface characteristics and optimizing the support materials to improve catalytic activity, stability, and overall effectiveness [88]. Surface engineering focuses on altering the composition, structure, and shape of the electrocatalyst surface to optimize electrochemical reactions at the electrode–electrolyte interface [146]. This can be achieved through surface functionalization, introducing active sites or functional groups to facilitate specific electrochemical reactions and improve reaction kinetics [146,147]. Additionally, surface modifications improve the interaction between the electrocatalyst and the electrolyte, enhance reactant and product transport, and minimize undesired side reactions [148]. For instance, through the utilization of a tellurium sacrificial template and surface customization, Agrawal et al. synthesized “pseudo-carbon nanotubes”. The ZIF-8 component was infused with a combination of iron, cobalt, and zinc to form CoFeZn@pCNTs that possess remarkable catalytic performance in ORR, evidenced by their E_1/2_ value equivalent to 0.87 V vs. RHE [77]. Catalyst support strategies, on the other hand, involve selecting and optimizing support materials that enhance electrocatalyst performance and stability. Carbon-based materials, such as carbon nanotubes, graphene, and carbon black, are commonly used as support materials due to their high surface area, which provides an enlarged active surface for catalytic reactions and improves conductivity [149]. In 2019, Jin and a team of researchers synthesized Fe, N, and S co-doped carbon nanotube nanocomposites (Fe-N-S CNN) by pyrolysis of ZIF-8 impregnated with iron salt. Here, the use of carbon-based materials when used as an electrocatalyst enhanced the ZAB performance showing a high specific capacity of 700 mA h g^−1^ [102]. Moreover, incorporating metal oxide nanoparticles or conducting polymers as support materials can also enhance catalytic activity and stability by shielding active sites from corrosion, providing additional catalytic sites, and improving structural integrity, including, for example, the use of conductive metallic foam such as nickel foam, copper foam, iron foam, nickel mesh, etc. [145,150]. Through the adoption of an in situ growing technique, Xiong et al. successfully created NiFe MOF, NiCo MOF, Fe MOF/Ni MOF, and Co MOF/Ni MOF. The Ni Foam served as a conductive substrate during the electrocatalytic reaction to enhance electron transfer while also preventing MOF structure collapse [151]. Likewise, in 2020, Chen and a team of researchers synthesized a composite (Co-NC@LDH) by anchoring NiFe-layered double hydroxide (NiFe-LDH) nanosheets on the surface of a ZIF-derived carbon-based framework. The obtained electrocatalyst showed impressive catalytic efficiency, great kinetics, and adequate durability against OER due to the extensively exposed metal active sites, improved conductivity, and stable structure [25]. Thus, it can be said that surface engineering and catalyst support strategies offer promising ways for developing efficient and durable electrocatalysts for zinc–air batteries.

## 6. Challenges and Future Perspectives

Zinc–air batteries face challenges in terms of limited cycle life, dendrite formation, air electrode stability, electrolyte degradation, and scalability [152,153]. To enhance their performance, research focuses on developing advanced electrode materials and electrolytes to prevent dendrite formation and improve cycle life [154]. Improving the catalytic activity, durability, and stability of catalyst materials in the air electrode is crucial. Stable and efficient electrolyte formulations are needed to minimize side reactions and ensure high ionic conductivity [155]. Scalable and cost-effective manufacturing processes are essential for commercial viability. Future research should explore new materials, designs, and technologies to address these challenges and integrate zinc–air batteries with renewable energy sources and smart grids. Continued research and development will unlock the potential of zinc–air batteries for energy storage applications.

### 6.1. Limitations and Challenges in Using (Fe-Co-Ni-Zn)-Based MOFs-Derived Electrocatalysts

The utilization of (Fe-Co-Ni-Zn)-based MOFs-derived electrocatalysts encounters certain limitations and challenges. One limitation is the instability of MOFs in aqueous environments, leading to degradation and reduced catalytic activity over time [156]. This poses a difficulty in maintaining the electrocatalysts’ performance over extended durations. Additionally, the relatively low electrical conductivity of MOFs hampers efficient charge transfer during electrochemical reactions, affecting overall efficiency [157]. Another challenge lies in the complex and costly large-scale synthesis of MOFs with desired properties. Cost-effective and scalable synthesis techniques are necessary to overcome this obstacle. Ongoing research focuses on optimizing synthesis methods, enhancing electrical conductivity, improving stability, and enhancing selectivity to enhance the performance and applicability of (Fe-Co-Ni-Zn)-based MOFs-derived electrocatalysts for diverse energy storage and conversion applications.

### 6.2. Potential Solutions and Future Research Directions

To address the limitations and challenges associated with (Fe-Co-Ni-Zn)-based MOFs-derived electrocatalysts, several potential solutions and future research directions can be explored. One strategy is to enhance the stability of MOFs in aqueous environments by implementing protective coatings or encapsulation techniques. This would prevent degradation and maintain catalytic activity over extended periods. Another approach is to improve the electrical conductivity of MOFs by incorporating conductive materials or introducing dopants. This would facilitate more efficient charge transfer and enhance overall electrocatalytic performance. Additionally, scalable and cost-effective synthesis methods need to be developed for large-scale production, which may involve optimizing reaction conditions, exploring alternative precursors, or implementing continuous flow synthesis approaches. Increasing the selectivity of MOF-derived electrocatalysts can be achieved by tailoring active sites or introducing functional groups to minimize unwanted side reactions. Long-term stability and durability under harsh conditions can be addressed by designing more robust catalyst architectures. Integration of MOF-derived electrocatalysts with other materials or catalyst supports could lead to synergistic effects and improved performance. This could involve exploring carbon-based nanomaterials or metal oxides as catalyst supports to enhance stability and promote efficient charge transfer. Overall, future research should focus on advancing the stability, electrical conductivity, scalability, selectivity, and durability of (Fe-Co-Ni-Zn)-based MOFs-derived electrocatalysts through innovative synthesis methods, structural modifications, integration with other materials, and advanced characterization techniques. These efforts will contribute to the development of highly efficient and practical electrocatalysts for various energy storage and conversion applications.

### 6.3. Emerging Trends and Opportunities

Zinc–air batteries are currently emerging trends and opportunities that have the potential to revolutionize the field of energy storage. These include advancements in rechargeability, the development of innovative electrolyte solutions, catalyst design and engineering, miniaturization for micro-power applications, grid-scale energy storage, and a focus on environmental sustainability [11,93]. Efforts are being made to enhance the rechargeability of zinc–air batteries by addressing challenges such as dendrite formation and electrode degradation [158,159]. Novel electrolyte solutions are being explored and optimized to improve battery efficiency and stability. Catalyst design is also a key area of research, aiming to create efficient and long-lasting catalysts that can enhance electrochemical reactions [160]. The miniaturization of zinc–air batteries enables their integration into small devices like wearables and IoT devices. Furthermore, the high energy density and cost-effectiveness of zinc–air batteries make them attractive for grid-scale energy storage applications. Environmental benefits, including the use of non-toxic and abundant materials, further contribute to the appeal of zinc–air batteries. Ongoing research and development efforts in these areas are expected to drive the commercialization and widespread adoption of zinc–air batteries across various industries and applications.

## 7. Conclusions

Zinc–air batteries (ZABs), due to their high energy density, cost-effectiveness, and environmental friendliness, are appealing for their use in renewable energy storage, electric vehicles, and various other applications. Electrocatalysts play a vital role in enhancing their performance by facilitating the ORR and OER, reducing energy losses, improving reaction kinetics, and increasing their specific capacity. The present study provided a review of electrocatalysts derived from Fe, Co, Ni, and Zn-based MOFs and their use in ZABs. They can be synthesized using various techniques, including solvothermal, hydrothermal, microwave-assisted, and electrochemical methods. Their structure, morphology, composition, and electrochemical properties are provided by several characterization methods, which include XRD, SEM, TEM, EDS, FTIR, XPS, and electrochemical techniques. The (Fe-Co-Ni-Zn)-based MOF-derived electrocatalysts offer promising properties such as abundance, catalytic activity, tunability, and structural stability. These electrocatalysts exhibit high ORR and OER activity, stability, selectivity, and a large surface area, making them attractive for energy storage and conversion applications. In zinc–air batteries, they contribute to higher energy density, cell efficiency, and a longer lifespan. Further research and development are crucial to optimizing their performance and enabling the widespread use of ZABs in various fields.

The main findings and contributions of the study involve the successful development of MOFs utilizing iron, cobalt, nickel, and zinc. These MOFs demonstrate exceptional electrocatalytic capabilities for zinc–air batteries, resulting in improved energy density and catalytic performance. This review provides an idea for enhancing the affordability and effectiveness of zinc–air battery technology, thereby advancing the field of energy storage solutions.

The importance and potential of (Fe-Co-Ni-Zn)-based MOFs-derived electrocatalysts in zinc–air batteries lie in their ability to enhance battery performance and affordability. These electrocatalysts, derived from MOFs, exhibit impressive electrocatalytic properties, leading to improved energy density and catalytic efficiency in zinc–air batteries. Thus, zinc–air battery research offers promising prospects for advancing more efficient and cost-effective energy storage solutions, making it a valuable contribution to battery technology.

## Figures and Tables

**Table 1:** Electrocatalytic and ZAB performances shown by oxygen electrocatalysts of (Fe-Co-Ni-Zn)-based MOF materials.

S. N.	Electrocatalysts	ORR E_onset_ [V]	ORR E_1/2_ [V]	Open Circuit Voltage [V]	Specific Capacity [mAh g^−1^]	Durability @mA cm^−2^	Peak Power Density [mW cm^−2^]	Ref.
1.	Fe-N-CNT	1.015	0.89	1.36	720	-	131.7	[24]
2.	ZFN-900	-	0.85	1.21	-	-	115.8	[26]
3.	Ni_0.6_Fe_0.4_CM	0.88	0.75	1.44	-	69 h @ 10	59.83	[100]
4.	Co_5.47_N/Co_3_Fe_7_/NC	-	0.89	1.502	-	180 h @ 5	264	[22]
5.	3DOM Fe-N-C	-	0.875	1.45	768.3	100 h @ 5	235	[101]
6.	NiFe-MOF/NiFe_2_O_4_	0.73	-	1.39	700	-	158.4	[62]
7.	Fe-N-S CNN	-	0.91	1.37	700	-	132	[102]
8.	FeCu-BTC/WO_3_-WC	-	0.81	1.43	-	300 h @ 5	135.2	[20]
9.	Co-NC@LDH	-	0.80	1.41	806	300 h @ 5	107.8	[25]
10.	(Zn,Co)/NC	-	0.87	1.2	807	60 h @ 5	186	[103]
11.	Zn/Mo2C@Co-NCNTs	0.918	0.838	1.506	741.9	100 h @ 0.5	223.54	[104]
12.	CoZn-NCNTs	0.94	0.82	1.46	757	320 h @ 2	214	[105]
13.	ES-Co/Zn-CN_ZIF_	0.9953	0.857	1.369	802.6	254 @ 10	42.37	[106]
14.	Co-MOF-800	-	0.84	1.38	671.6	54 @ 10	144	[107]
15.	FeS/Fe_3_C@NS-C-900	1.03	0.78	1.455	750	865 h @ 2	90.9	[108]
16.	Co_0.25_Ni_0.75_@NCNT	0.94	0.84	1.53	-	36 h @ 5	167	[109]
17.	FeCO_3_−NC-1100	1.05	0.877	2.958	-	190 h @ 10	372	[110]
18.	Mn/Co-N-C-0.02-800	0.90	0.80	1.39	-	120 h @ 20	136	[111]
19.	Co-N-CNT	0.97	0.90	1.365	-	15 h @ 2	101	[112]
20.	FeNiCo@NC-P	-	0.84	1.36	807	130 h @ 10	112	[56]
21.	CoP_x_@CNS	0.83	0.76	1.40	-	130 h @ 5	110	[113]
22.	Co_x_P@NPC	-	0.82	1.43	-	140 h @ 5	157	[114]
23.	FeNiP/NCH	-	0.75	1.48	-	500 h @ 10	250	[115]

## Data Availability

Not applicable.
